# Senescence induces miR‐409 to down‐regulate CCL5 and impairs angiogenesis in endothelial progenitor cells

**DOI:** 10.1111/jcmm.18489

**Published:** 2024-06-20

**Authors:** Yen‐Hung Chou, Yi‐Nan Lee, Cheng‐Huang Su, Hsin‐I Lee, Chin‐Ling Hsieh, Ting‐Yi Tien, Chao‐Feng Lin, Hung‐I Yeh, Yih‐Jer Wu

**Affiliations:** ^1^ Department of Medicine MacKay Medical College New Taipei Taiwan; ^2^ Institute of Biomedical Sciences MacKay Medical College New Taipei Taiwan; ^3^ Division of Preventive Cardiology & Pulmonary Circulation Medicine, Department of Cardiovascular Medicine, Department of Internal Medicine and Department of Medical Research MacKay Memorial Hospital New Taipei Taiwan

**Keywords:** angiogenesis, CCL5, endothelial progenitor cells, miR‐409, senescence

## Abstract

This study explores the impact of senescence on autocrine C‐C motif chemokine ligand 5 (CCL5) in human endothelial progenitor cell (EPCs), addressing the poorly understood decline in number and function of EPCs during ageing. We examined the effects of replication‐induced senescence on CCL5/CCL5 receptor (CCR5) signalling and angiogenic activity of EPCs in vitro and in vivo. We also explored microRNAs controlling CCL5 secretion in senescent EPCs, its impact on EPC angiogenic activity, and validated our findings in humans. CCL5 secretion and CCR5 levels in senescent EPCs were reduced, leading to attenuated angiogenic activity. CCL5 enhanced EPC proliferation via the CCR5/AKT/P70S6K axis and increased vascular endothelial growth factor (VEGF) secretion. Up‐regulation of miR‐409 in senescent EPCs resulted in decreased CCL5 secretion, inhibiting the angiogenic activity, though these negative effects were counteracted by the addition of CCL5 and VEGF. In a mouse hind limb ischemia model, CCL5 improved the angiogenic activity of senescent EPCs. Analysis involving 62 healthy donors revealed a negative association between CCL5 levels, age and Framingham Risk Score. These findings propose CCL5 as a potential biomarker for detection of EPC senescence and cardiovascular risk assessment, suggesting its therapeutic potential for age‐related cardiovascular disorders.

## 1 INTRODUCTION

Endothelial progenitor cells (EPCs) have been proposed in cell therapy for ischemic diseases provoked by smoking, diabetes, hypertension and hyperlipidemia. Growing evidence indicates that the angiogenic and beneficial paracrine effects of transplanted EPCs play a crucial role in the treatment of ischemic diseases. Despite these promising results, general acceptance of EPCs for clinical therapies have remained hampered by several challenges.^1^ Ageing is an independent risk factor for the development of ischemic diseases, and EPC number is reduced along with the increase in age.^2^, ^3^, ^4^ Ageing results in a decline in progenitor cell angiogenic activities, including proliferation, migration and vessel tube formation.^5^, ^6^, ^7^ Ageing also impairs EPC recruitment and vascular incorporation.^8^ The available data indicate that ageing may affect the availability and angiogenic activity of EPCs. However, the underlying mechanisms of EPC senescence related dysfunction remain poorly understood.

CCL5 has been reported to facilitate tumour progression and metastasis. CCL5 is secreted from human osteosarcoma to induce EPC tube formation and migration.^9^ In tissue repair, EPCs migrate from bone marrow to tissues via the bloodstream to provide growth factors.^10^ Although the paracrine effects of EPCs had been widely described, production of CCL5 from EPCs themselves has never been addressed. Furthermore, the impacts of senescence on CCL5 secretion and EPC angiogenic activity are still unclear.

MicroRNAs (miRNAs) are small, single‐stranded, non‐coding RNAs which regulate gene expression at the post‐transcriptional level to fine tune physiological homeostasis.^11^, ^12^ MiRNAs directly bind the 3′ untranslated region (3′ UTR) of target messenger RNA (mRNA) to inhibit translation and/or promote the degradation of target mRNA.^13^ The expression of numerous miRNAs changes with age, suggesting that ageing‐induced alteration of miRNA biogenesis might have an important role in the development of ageing‐related diseases.^14^ It has been reported that dysregulated miRNAs in the process of endothelial cell senescence and the associated development of age‐related diseases.^15^ We had conducted global next generation sequencing (NGS) to investigate the differential expression of miRNAs in senescent and young EPCs. Expression of miR‐409 was identified in NGS screening and the underlying mechanisms for anti‐angiogenic effects were explored.^16^ In the present study, we examined the interaction between CCL5 and VEGF during EPC ageing and assessed the association of Framingham risk score (FRS) with CCL5 and VEGF in seniors' EPCs.

## 2 MATERIALS AND METHODS

### 2.1 Human EPC isolation and culture

Ethical approval (12MMHIS201) was granted by the Mackay Memorial Hospital Institutional Review Board, Taipei, Taiwan. Before the collection of peripheral bloods, informed consent was obtained from healthy donors. Eighty millilitre of peripheral bloods of healthy donors were collectted to isolate the peripheral blood mononuclear cells (PBMCs).^7^ PBMCs were fractioned from other components of bloods by Ficoll‐Paque™ plus (GE Healthcare, USA) gradient centrifugation. EPCs were isolated further using a CD34 MicroBead kit and MACS Cell Separation System (all from Miltenyi Biotec). CD34‐positive EPCs were grown on fibronectin‐coated dishes (BD Biosciences, USA) supplemented with the EGM‐2 BulletKit system (Lonza, Switzerland) consisting of endothelial basal medium, 2% fetal bovine serum (FBS), human epidermal growth factor, human fibroblast growth factor‐B, insulin‐like growth factor‐1, ascorbic acid and heparin and incubated in a 5% CO_2_ incubator at 37°C for 4 days. EPC colonies displayed cobblestone‐like morphology after culturing in MV2 medium for additional 10–14 days. Senescent EPCs were obtained after serial propagation and defined as the doubling time of more than two‐folds of the young EPCs.

### 2.2 Replication induces EPC senescence

EPCs below five passages from donors were defined as young EPCs, while the same clone with additional 10 passages was defined as old EPCs with cell doubling times (CDTs) reaching twice longer compared to the corresponding clones of young EPCs as previously described.^17^ To calculate CDT, 1 × 10^4^ cells were seeded on a 24‐well plate in parallel with each passage and cultured for 48 h. CDT was obtained using the following equation:

$$\mathrm{CDT}\;=\;\text{duration}\;\text{time}\;\times \;\log\;\left(2\right)/\log\left(1\;+\;r\right)$$

where *F* = final cell number and *I* = initial cell number, which was equal to 1 × 10^4^ in our setting. Growth rate, *r* = (*F*−*I*)/*I*. Duration time is 48 h.

### 2.3 Fluorescence imaging of SA‐β‐gal

Cell senescence was determined by measuring senescence associated β‐galactosidase (SA‐β‐gal) activity using SPiDER‐βGal kit (Dojindo, Kumamoto 861–2202, Japan). Cells were seeded at a density of 3 × 10^4^ cells per well in a 24‐well plate, fixed with 4% paraformaldehyde (PFA)/PBS at room temperature for 3 min, and washed three times with 1 mL of PBS. The SPiDER‐βGal DMSO stock solution was diluted 2000 times with McIlvaine buffer (citric acid, 7.4 mM; sodium phosphate, 1.2 mM; pH 6.0) and 1 mL of SPiDER‐βGal working solution was added to the cells followed by incubation at 37°C for 30 min. Nuclei were counter‐stained with DAPI for 5 min. After cells were washed 3 min with PBS, the images were acquired under Leica SP8 confocal microscopy using 488 nm excitation and 543 nm emission filters.

### 2.4 Cell growth and proliferation assay

EPCs (1 × 10^4^) were grown in a 24‐well plate with 500 μL of FBS (2%)‐containing MV2 medium. Cells were incubated with bromodeoxyuridine (BrdU) overnight according to manufacturer's instruction (EMD Millipore Corp., Billerica, MA, USA). Cells were fixed and incubated with a primary antibody against BrdU. A corresponding secondary antibody conjugated with horseradish peroxidase was applied to visualize BrdU by using tetra‐methylbenzidine as a substrate. Proliferation was measured with a SpectraMAX 190 absorbance microplate reader (Molecular Devices) at dual wavelengths of 450 and 540 nm.

### 2.5 Cell migration and tube formation assay

Cell migration assay was performed using a Boyden chamber to evaluate cell migrating rate in 24‐well transwell chambers (8 μm pore size, Corning). The transwell assay was performed in a chamber of two medium‐filled compartments separated by a microporous membrane. To evaluate the migration activity of EPCs (3 × 10^4^ cells in the upper compartment), 100 μL of serum‐free MV2 was placed in the upper compartment, while 600 μL of FBS (2%)‐containing MV2 was placed in the lower compartment. After incubation at 37°C for 4 h, the membrane between the two compartments was removed and fixed with ice‐cold ethanol (−20°C) for 5 min and stained with 18.7 μM bisbenzimide for 20 min (Sigma, St. Louis, MO, USA). The number of cells in the lower side of the membrane was determined by inverted fluorescence microscopy (Leica, Wetzlar, Germany) at 50× magnification and analysed using QWin image analysis software (Leica, Cambridge, England, UK, version number: V3.5.2).

For tube formation assay, cells were seeded at Matrigel (BD Biosciences) coated 24‐well plates at a density of 3 × 10^4^ cells per well. Cell images were acquired from four randomly selected microscopic fields at 50× magnification derived from three independent experiments. The cumulative tube length was calculated using QWin as an indicator of their angiogenic potential.^18^

### 2.6 MiRNAs transfection

EPCs were grown on 1% gelatin‐coated petri dishes and kept in the range of 70 to 80% confluency for transfection experiments. All miRNAs and nonsense controls were transfected using Lipofectamine 3000 (Invitrogen). Commercial products of miR‐409‐3p mimic (# MC12446) and mimic negative control (#4464058) were purchased from Thermo Fisher Scientific Inc. After 5.5 h of incubation at the time of transfection, miRNA mixtures were replaced with 20% FBS MV2 for recovery.

### 2.7 Enzyme‐linked immunosorbent assay

EPCs were transfected with miR‐409 for 48 h. The culture supernatants of EPCs were collected, centrifuged and stored at −80°C. CCL5 and VEGF in each supernatant were quantified using human quantikine ELISA kits (R&D Systems, Minneapolis, MN, USA) according to the recommended procedures. Optical density was measured with a SpectraMAX 190 absorbance microplate reader (Molecular Devices, San Jose, CA, USA) at 450–540 nm.

### 2.8 Quantitative reverse transcription‐PCR

Total RNAs were extracted from EPCs using the RNeasy Plus mini kit (Qiagen, Germany). The first strand cDNA was synthesized from 1 μg of RNA using the SuperScript III First‐Strand Synthesis System kit (Invitrogen, USA). Real‐time PCR was amplified with primers specific for CCL5, CCR5 and β‐actin using iQ SYBER Green Supermix reagent and detected with the iQ single‐colour real‐time PCR detector system (all from Bio‐Rad, USA).

CCL5 sense strand primer: 5′‐AGATCTCTGCAGCTGCCCTCA‐3′.

CCL5 antisense strand primer: 5′‐GGAGCACTTGCTGCTGGTGTAG‐3′.

CCR5 sense strand primer: 5′‐GGTTCCTGAAAGCGGCTGTAAATA‐3′.

CCR5 antisense strand primer: 5′‐CTGTTGGCAGTCAGGCACATC‐3′.

β‐actin sense strand primer: 5′‐CACCATTGGCAATGAGCGGTTC.

β‐actin antisense strand primer: 5′‐AGGTCTTTGCGGATGTCCACGT.

Data were analysed with iQ5 optical system software, Version 2.0 (Bio‐Rad). Relative mRNA levels were normalized with the corresponding levels of β‐actin.

### 2.9 Western blotting analysis

EPCs were lysed with RIPA buffer supplemented with protease inhibitor (Roche 11836170001) and protein concentrations were determined by modified Lowry's method. Aliquots of cell lysates were loaded into 12% SDS‐polyacrylamide gels, electrophoresed and transblotted onto nitrocellulose membranes (Millipore). The blots were blocked with 10% bovine serum albumin for 1 h and probed with indicated primary antibody for 2 h. The blots were further incubated with alkaline phosphatase‐conjugated secondary antibodies for 1 h at room temperature. Immunoreactivity was visualized using CDP‐star system (Roche) according to the manufacturer's instruction. Primary antibody for CCR5, AKT, p‐AKT, P70S6K, p‐P70S6K and GAPDH (all from Cell signalling) were diluted with PBS in 1 to 1000.

### 2.10 Animal experiments

All animal experiments and protocols were approved by the Institutional Animal Care and Use Committee of the MacKay Memorial Hospital (approval number: MMH‐A‐S‐106‐35). Experiments were performed on female 8‐week‐old athymic BALB/c mice (National Laboratory Animal Center, Taiwan) maintained under a 12‐h light/dark cycle. Standard laboratory chow and water were available ad libitum.

### 2.11 Angiogenesis model using hindlimb ischemia mice

To create ischemic hindlimbs, 8‐week old nude mice (female athymic BALB/c mice, weighed 18–22 g) were cut off the right femoral artery and vein, from just above the deep femoral arteries to the popliteal artery and vein, with anaesthesia induced by intraperitoneal injection of pentobarbital (80 mg/kg). Twenty‐four hours after surgery, right thighs and calves of animals were injected with one of the following solutions: (1) 50 μL PBS alone (PBS Group); (2) PBS containing 0.1 ng/mL of CCL5 (CCL5 Group); (3) PBS containing old EPC (2 × 10^5^) (OEPC Group); (4) PBS containing old EPC (2 × 10^5^) with 0.1 ng/mL of CCL5 (CCL5 + OEPC Group). Twenty‐one days later, animals were sacrificed by intraperitoneal injection of pentobarbital (100 mg/kg). Calf muscles were harvested and processed for immunohistochemical analysis.

### 2.12 Laser Doppler perfusion imaging

The perfusion rates of hind limbs were measured using a Laser Doppler Imager (Moor Instruments, Milwey, United Kingdom). Mice were anaesthetised with intraperitoneal injection of pentobarbital (80 mg/kg) and kept warm on a heater at 37°C for 10 min before scanning the hind legs and feet. Laser Doppler imaging of perfusion of subcutaneous hindlimbs was acquired at 24 h after surgery (just before the injection of EPCs), and then repeated on Days 7, 14 and 21 post EPC injection, with perfusion status expressed as a ratio of right (ischemic) to left (normal) hindlimb.^18^

### 2.13 Immunohistochemistry

Calf muscles were fixed with 4% paraformaldehyde at room temperature for 10 min, followed with 30% sucrose‐PBS at 4°C for 24 h. Calf muscles were bisected at the middle level, mounted in OCT compound (Leica) and snap‐frozen in liquid nitrogen. Capillary density was determined by bisecting calf muscle with 3 mm apart from each other. Both sections fixed with methanol for 10 min and washed briefly with PBS. After blocking with 10% horse serum, tissues were labelled with a mixture of a monoclonal rat anti‐murine platelet‐endothelial cell adhesion molecule‐1 (CD31) antibody (1:200; B&D Pharmingen, San Diego, CA) and a polyclonal rabbit anti‐laminin antibody (1:100; Chemicon) at 37°C for 2 h followed by incubation with a mixture of CY3‐conjugated anti‐rat antibody and CY5‐conjugated anti‐rabbit antibody (both from Chemicon). In parallel, methanol‐fixed frozen sections were stained with TRITC‐conjugated murine EC‐specific Bandeiraea simplicifolia lectin 1 (1:50 Sigma) and a polyclonal rabbit anti‐laminin antibody (1:100; Chemicon) at 4°C overnight followed by incubation with a CY5‐conjugated anti‐rabbit antibody. Capillaries were counted per 30 randomly chosen high‐power fields on the two sections per animal (without amputated hindlimb). The results were expressed as capillaries per myocyte.

### 2.14 Statistical analysis

Continuous data were expressed as the mean ± standard deviation and compared through a Student's *t*‐test. Categorical data were presented as a ratio and compared using a chi‐square test or Fisher's exact test. The difference among means for three or more groups was assessed by ANOVA. A linear regression or curve fitting test was used to assess the correlation between two variables. All *p*‐values were two‐sided, and a value less than 0.05 was considered as significant difference.

## 3 RESULTS

### 3.1 Senescence inhibits the secretion of CCL5, which enhances angiogenic potential of EPCs

**FIGURE 1:**
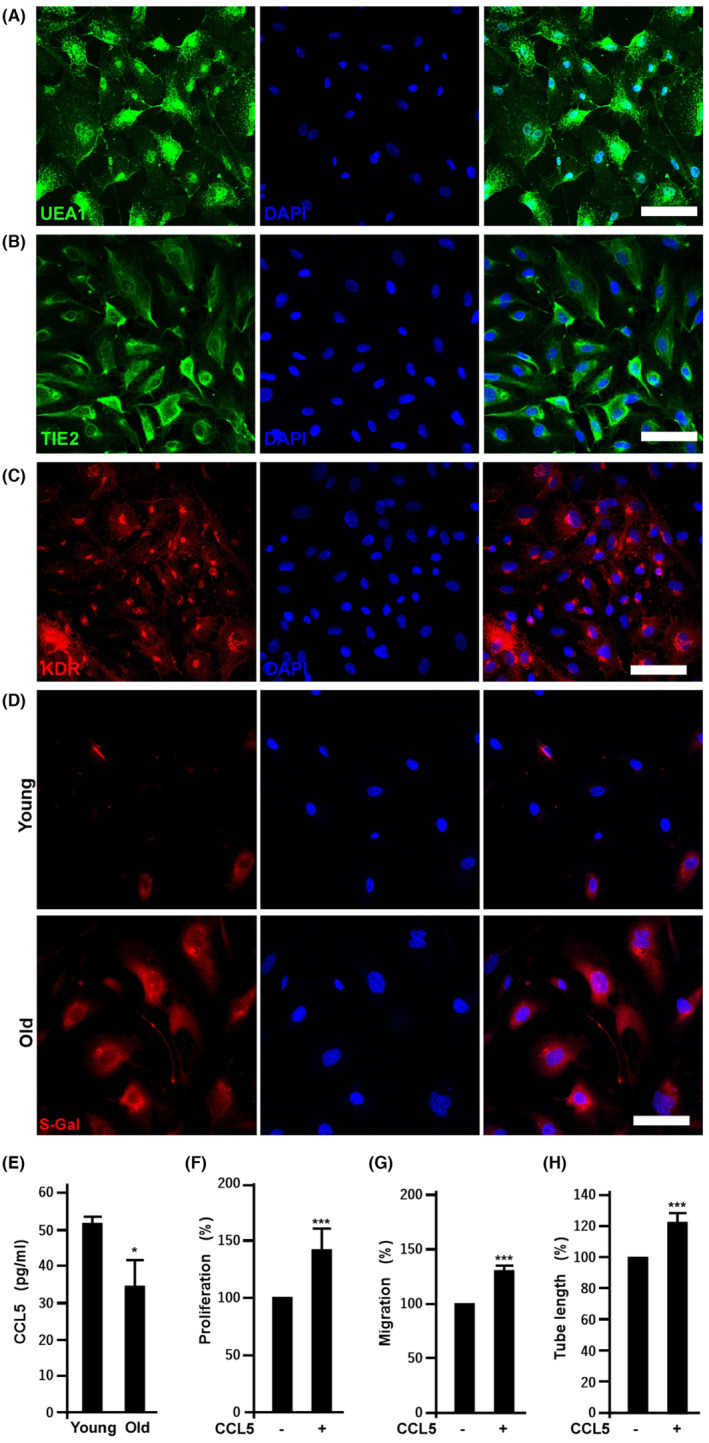
Senescence inhibits the secretion of CCL5, which enhances angiogenic potential of EPCs. (A) to (C) Effects of 10 serial passages on the expression of EPC markers, UEA1, TIE2 and KDR. EPCs below five passages from donors were defined as young EPCs, while the same cell line with additional 10 passages was defined as old cells. (D) Fluorescence imaging of SA‐β‐gal activity in young and old EPCs. (E) Effects of replication‐induced senescence on CCL5 secretion. CCL5 was harvested and quantified from the supernatants of young and old EPCs. Versus young EPCs, *, *p* < 0.05, *N* = 3. (F), (G) and (H), CCL5 regulates old EPC proliferation, migration and tube formation, respectively. Cells were treated without and with 0.1 ng/mL of CCL5 (#SRP3269, Sigma‐Aldrich). Cell proliferation was determined by BrdU incorporation, migration measured by transwell assays, and tube formation assay using Matrigel. Values are mean ± SD of triplicate assays from three independent experiments. **, *p* < 0.01; ***, *p* < 0.001 versus untreated cells.

EPCs isolated from PBMCs were characterized by the presence of endothelial cell markers including UEA1, TIE2 and KDR (Figure 1A,C), consistent with our previous studies^7^, ^18^ EPC senescence was induced by undergoing additional 10 serial passages, resulting in an increase in doubling time. To confirm whether the increased doubling time by serial passages contributes to cell senescence, SA‐β‐gal activity was monitored by incubating cells with non‐fluorescence SPiDER‐βGal substrates *in situ*.^19^ As Figure 1D shown, SA‐β‐gal activity was readily detected in old EPCs compared to vague signals of young cells, consistent with the previous findings.^7^

CCL5 plays a critical role in establishing environmental factors for age‐associated myeloid skewing phenotypes and is a pivotal chemokine for EPC homing,^20^ and the impact of senescence on the expression of CCL5 in EPC levels is still unclear. Here we compared CCL5 levels in young and old EPCs. As shown in Figure 1E, the secretion of CCL5 is dramatically decreased in senescent (Old) EPCs. The role of CCL5 on old EPC angiogenic activity was examined by proliferation, migration and tube formation assays. As shown in Figure 1E–H, exogenous CCL5 promotes EPC angiogenic potential.

### 3.2 CCL5 regulates EPC angiogenic potential via CCR5/AKT/P70S6K axis

**FIGURE 2:**
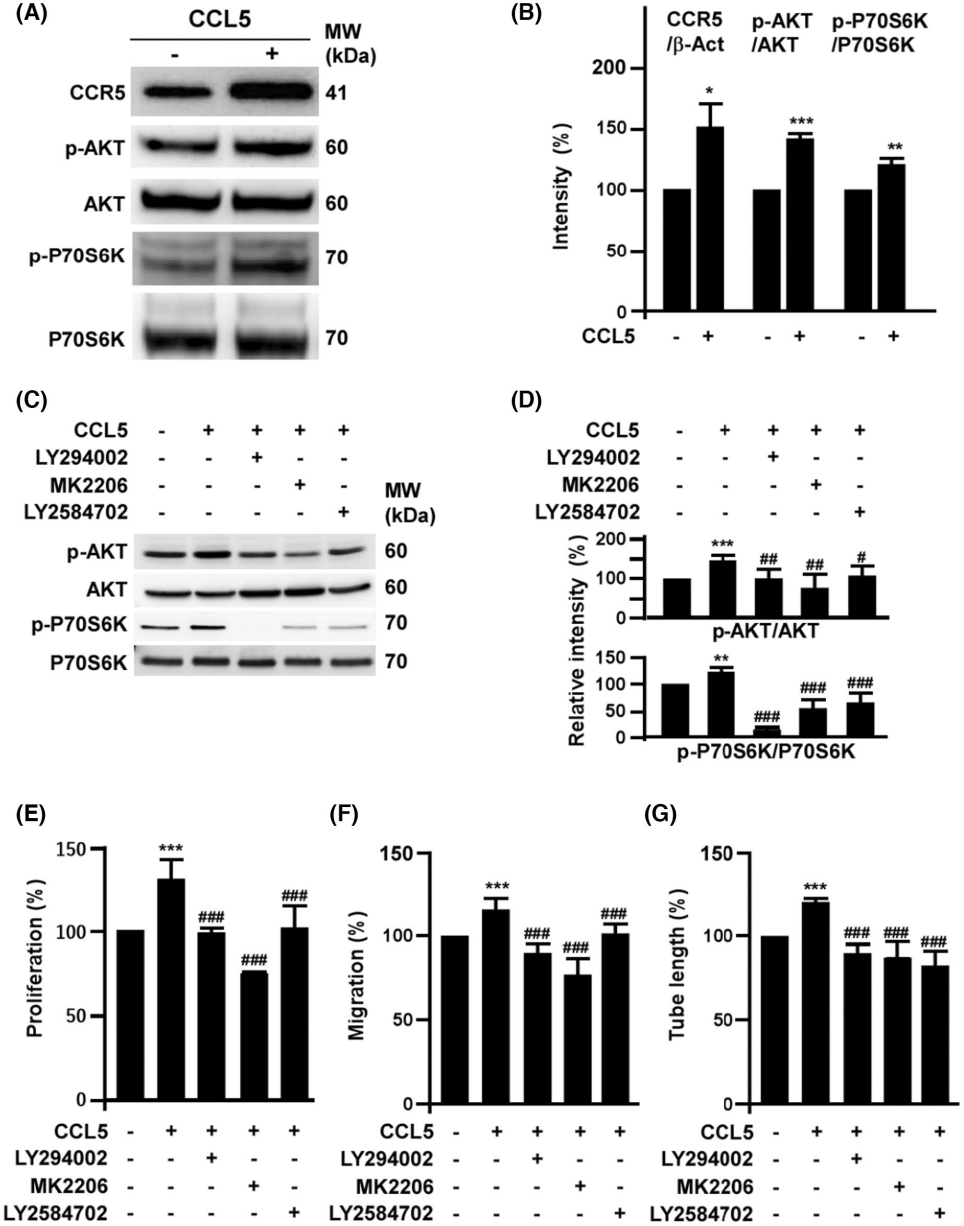
CCL5 regulates EPC angiogenic potential via CCR5/AKT/P70S6K axis. (A) Effects of CCL5 on the activation of CCR5/AKT/P70S6K axis. EPCs were treated with 0.1 ng/mL of CCL5 for 24 h then harvested to detect the expression of CCR5, total and phosphorylated AKT as well as P70S6K by Western blot assay. (B) Quantification of triplicate Western blot assays from three independent experiments of the activation of CCR5/AKT/P70S6K. (C) Effects of LY294002, MK2206 and LY2584702 on CCL5‐mediated AKT/P70S6K activation. Cells were pretreated with LY294002 (2 μM), MK2206 (0.5 μM) and LY2584702 (1 μM) overnight followed by CCL5 (0.1 ng/mL) treatment for additional 24 h. Quantification results were shown in (D). (E) to (G), effects of CCR5/AKT/P70S6K activation on CCL5‐mediated EPC angiogenic potential. The relative proliferation and migration rates were measured after cells treated with 0.1 ng/mL of CCL5 for 18 h. EPC proliferation was determined by BrdU incorporation while migration was measured by transwell assays. For tube formation assay, EPCs were seeded on Matrigel. Values are mean ± SD of triplicate assays from three independent experiments. *, Compared with untreated cells; #, compared with CCL‐5 treated group. ** and ##*, *p* < 0.01; *** and ###, *p* < 0.001.

It has been reported that AKT/P70S6K axis determines the angiogenic activity of EPCs.^21^, ^22^, ^23^ In the present study, CCL5 induced AKT/P70S6K activation and increased the expression of CCR5 in EPCs (Figure 2A,B). Activation of AKT and P70S6K were inhibited by MK2206 and LY2584702, respectively (Figure 2C,D). In angiogenesis assay, all PI3K, AKT and P70S6K were required for CCL5‐mediated angiogenic activity (Figure 2E–G).

### 3.3 Senescence induces miR‐409 to down‐regulate CCL5/CCR5 level leading to angiogenic inhibition

**FIGURE 3:**
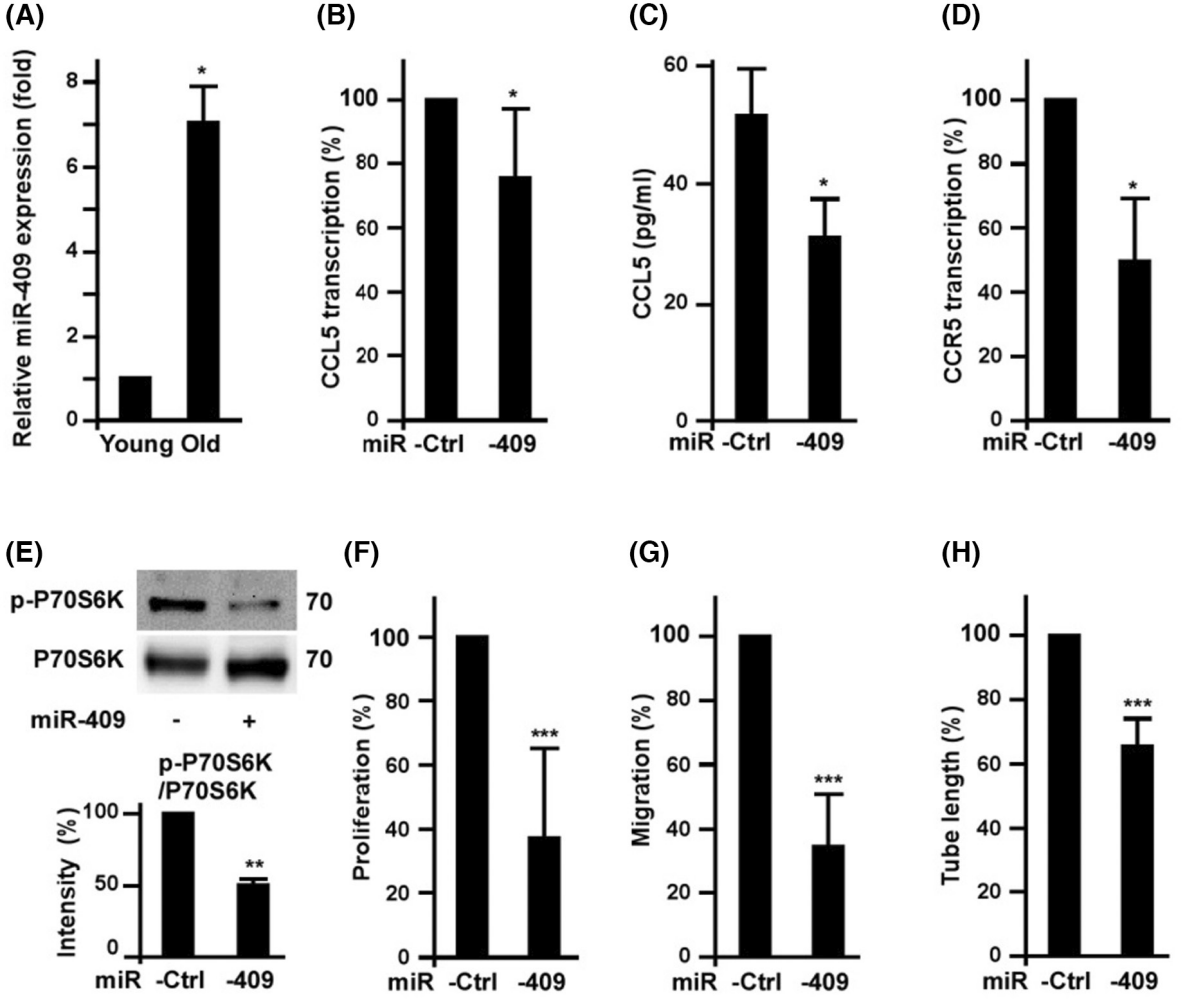
Senescence induced miR‐409 to down‐regulate CCL5/CCR5 level and decrease EPC angiogenic potential. (A) The effects of replication‐induced senescence on the expression of miR‐409 in EPCs. Young and old EPCs were harvested for miR‐409 measurement by quantitative PCR. *, *p* < 0.05, versus young EPCs. *N* = 3 (B) to (D) Effects of ectopic expression of miR‐409 on the level of CCL5 and CCR5. Cells were harvested after 2 days of miR‐409 or miR‐control transfection. Transcription of CCL5 and CCR5 were measured by quantitative RT‐PCR. For CCL5 levels, conditioned media of the EPCs were harvested to determine CCL5 by ELISA kit (C). *, *p* < 0.05, vs. Ctrl (miR‐control). (E) Effects of miR‐409 on CCL5‐induced P70S6K activation. EPCs were transfected with miR‐409 or miR‐control for 2 days, followed by CCL5 treatment overnight. Cells were harvested for Western blot assay. **, *p* < 0.01 (F) to (H), Effects of miR‐409 ectopic expression on EPC angiogenic potential. Cell proliferation and migration were measured after 2 days of miR‐409 or miR‐control transfection. ***, *p* < 0.001, versus miR‐control.

Consistent with our previous global NGS results of miRNAs in young and senescent EPCs,^16^ the up‐regulation of miR‐409 in old EPCs identified in the NGS screening was confirmed by qPCR (Figure 3A). Ectopic expression of miR‐409 significantly decreased the transcription of CCL5 and CCR5 as well as the translation of CCL5 (Figure 3B–D). As expected, miR‐409 abrogates CCL5‐induced P70S6K activation (Figure 3E). Ectopic expression of miR‐409 profoundly decreased EPC proliferation, migration as well as tube formation (Figure 3F–H).

### 3.4 CCL5 increases VEGF level to counteract the suppressive effect of miR‐409 on EPC angiogenic potential

**FIGURE 4:**
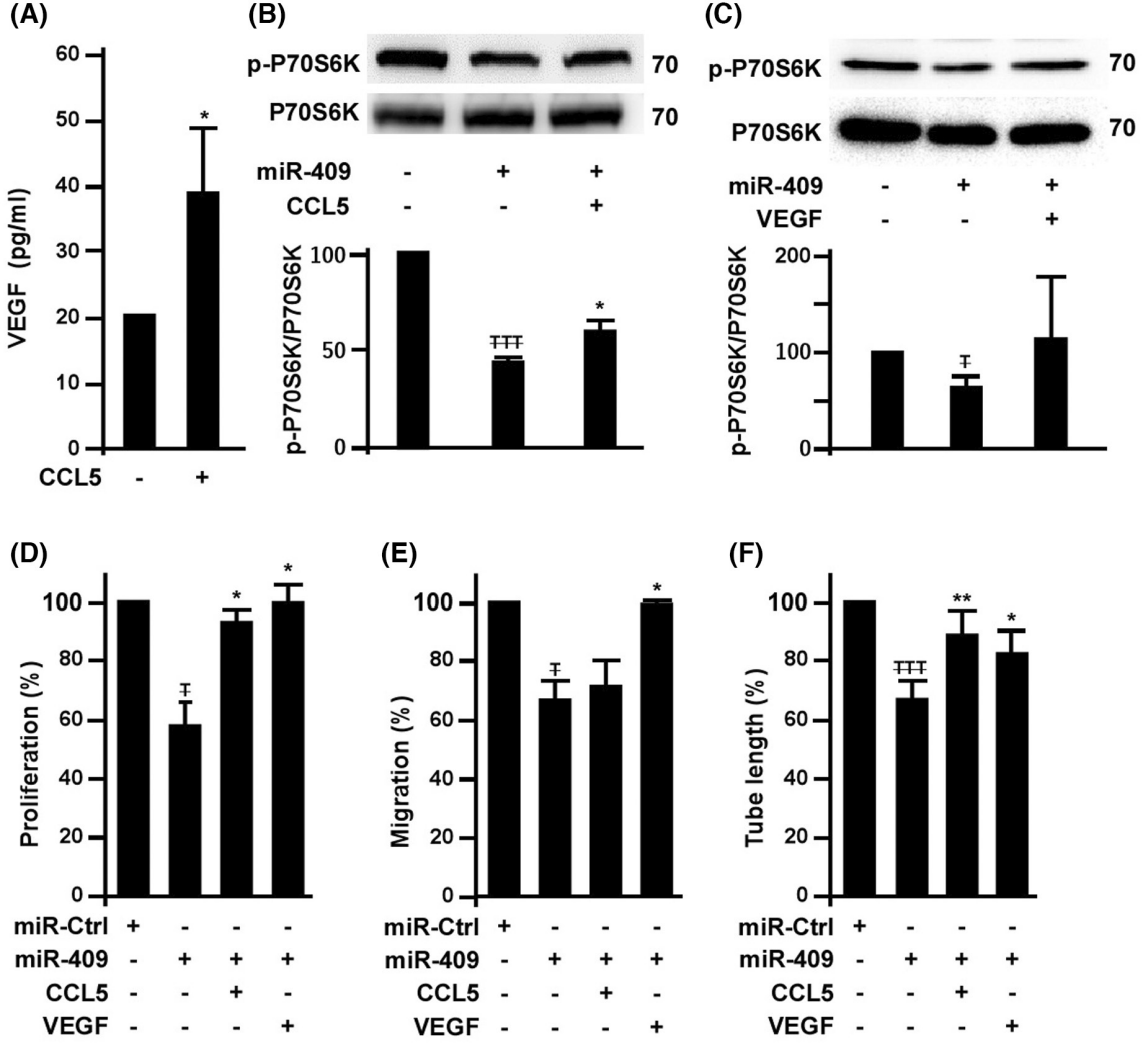
CCL5 increases VEGF level to counteract the suppressive effect of miR‐409 on EPC angiogenic activity. (A) Effects of CCL5 on VEGF secretion. EPCs were treated with 0.1 ng/mL of CCL5 for 24 h and the supernatants were harvested for VEGF concentration measurement. *, *p* < 0.05. (B) Effects of CCL5 on miR‐409‐mediated P70S6K inhibition. EPCs with 2 days of miR‐409 transfection were cultured with CCL5 (0.1 ng/mL). After CCL5 treatment overnight, cell lysates were harvested for Western blot analysis. ^T^, compared with untreated cells; *, compared with CCL‐5 treated group. ^TTT^, *p* < 0.001; *, *p* < 0.05. (C) Effects of VEGF on miR‐409‐mediated P70S6K inhibition. EPCs post 2 days of miR‐409 transfection were cultured with 50 ng/mL of VEGF for additional 16 hrs. ^T^, *p* < 0.05. (D) to (F), Effects of CCL5 and VEGF on miR‐409‐mediated angiogenic inhibition. EPCs post 2 days of miR‐409 or miR‐control transfection were cultured with CCL5 (0.1 ng/mL) and VEGF (50 ng/mL). Angiogenic potential assays were performed in cells treated with CCL5 and VEGF overnight. ^T^, compared with untreated cells; *, compared with miR‐409 only group. ^T^ and *, *p* < 0.05.

We tested whether CCL5 counteracts the anti‐angiogenic effects of miR‐409. Indeed, CCL5 stimulated VEGF secretion from EPCs (Figure 4A), consistent with previous studies in human chondrosarcoma cells.^24^ In addition, inhibition of P70S6K activation by ectopic expression of miR‐409 was resumed partially by CCL5 and almost totally by VEGF treatment (Figure 4B,C). Functional assay showed that the suppressive effects of miR‐409 on EPC angiogenic potential were blunted by CCL5 and VEGF treatment (Figure 4D–F).

### 3.5 CCL5 improves the angiogenic activity of senescent EPCs in hind limb ischemic tissues

**FIGURE 5:**
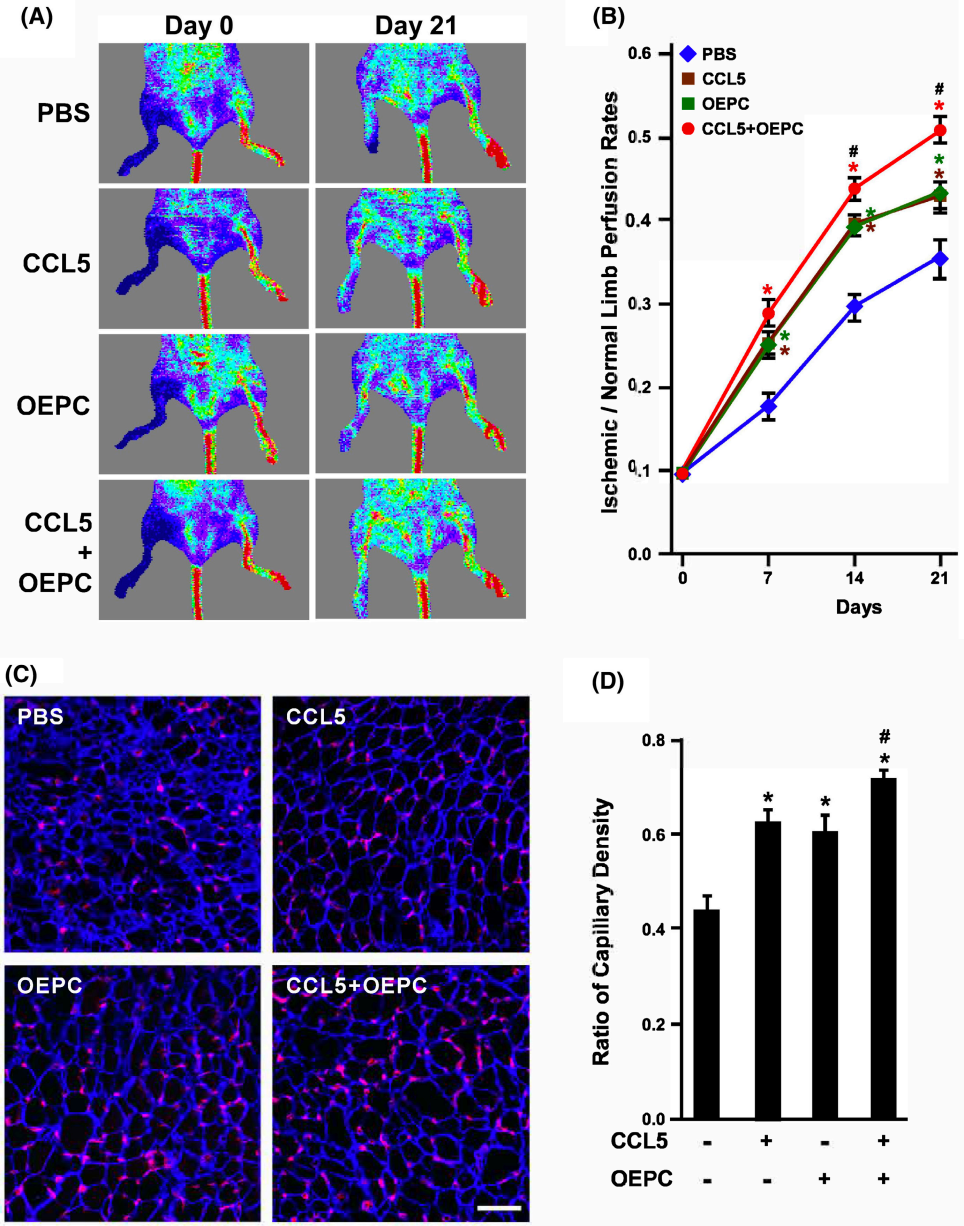
Effects of CCL5 on old EPC angiogenic ability in vivo. (A) Laser‐Doppler perfusion imaging of mouse hind limbs injected with human old EPCs (OEPC). Right hind limbs were injected with PBS, CCL5, OEPC and CCL5 combined with OEPC, and imaged at Day 0 and Day 21. Left hind limbs were untreated as control. (B) Quantification of hind limb perfusion rates. The area of ischemia versus normal perfusion were quantified. The days of imaging were as indicated. *, *p* < 0.05 compared with the PBS group; #, *p* < 0.05 compared with the OEPC group. (C) Ischemic hind limbs were sectioned to examine capillary densities at Day 21. Tissues were stained with laminin (blue) and lectin1 (red) to visualize myocytes and capillaries, respectively. (D) Quantification of capillary density of ischemic hind limbs. Capillary density was defined as the number of capillaries divided by the number of myocytes. *, *p* < 0.05 compared with the PBS (untreated) group; #, *p* < 0.05 compared with the OEPC group.

To confirm the effects of CCL5 on angiogenic ability of senescent EPC in vivo, mouse ischemic hind limbs were injected with human old EPCs (OEPC). The hind limb perfusion rates were improved in animals with CCL5 or OEPC injection (Figure 5A,B). More importantly, the perfusion rates were synergistically increased further in the CCL5 combined with OEPC group, suggesting that CCL5 enhanced the angiogenic activity of OEPC to further improve the perfusion rates. The ischemic tissues were sectioned to examine their capillary densities. Consistently, CCL5 or OEPC injection increased capillary density which was increased further in the CCL5 combined with OEPC group (Figure 5C,D).

### 3.6 Low levels of CCL5 and VEGF in seniors' EPCs are associated with higher Framingham risk score (FRS)

**FIGURE 6:**
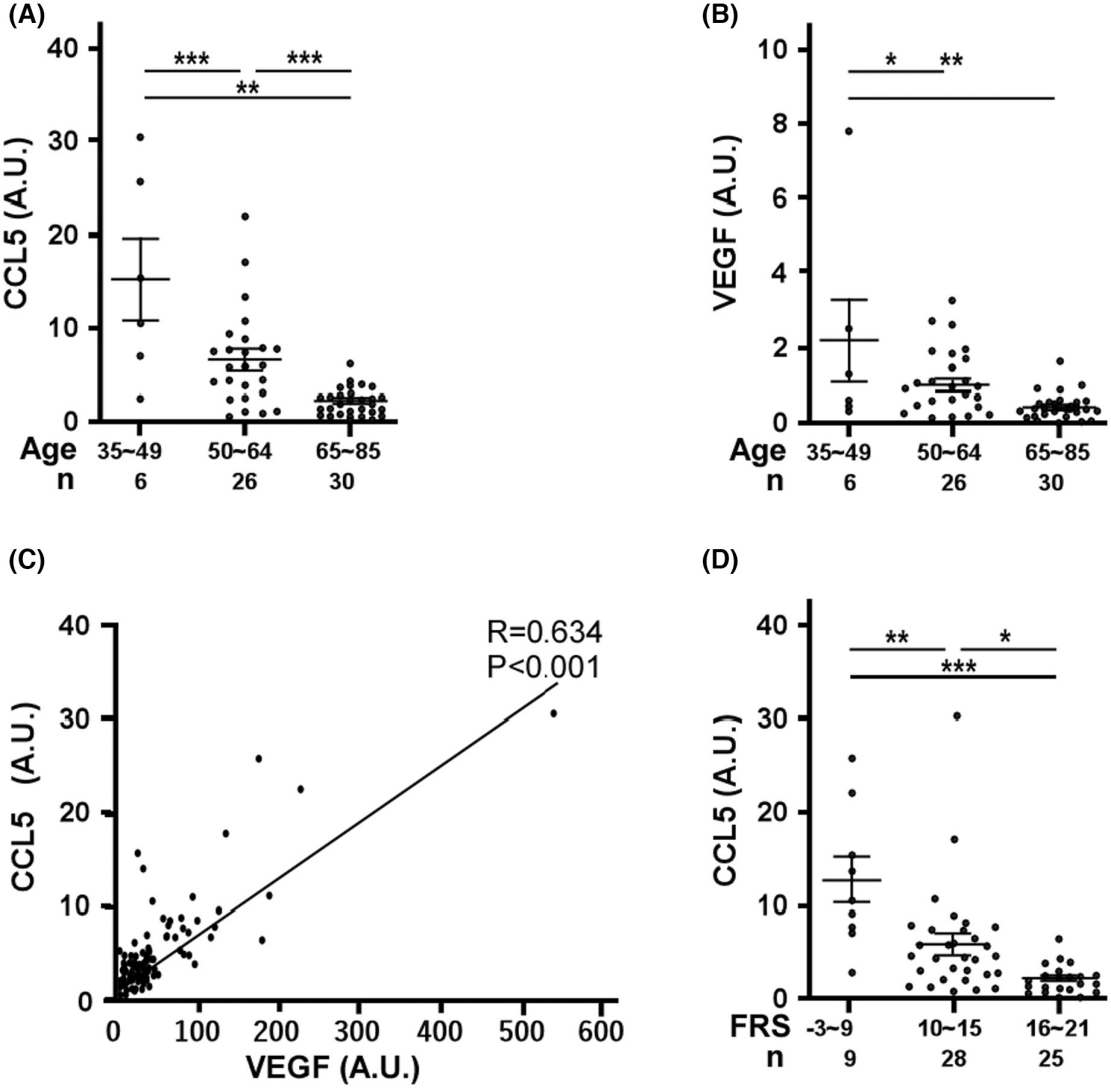
The relationships among CCL5‐ or VEGF‐secreting capacity, age and Framingham score. Circulating EPCs harvested from donors' peripheral bloods were cultured for 4 days to measure the concentrations of CCL5 and VEGF. (A) Scatter plot of VEGF (A.U.) versus donor's age. A.U. is defined as pg/mL/average cell number from four randomly selected microscopic fields at 50× magnification. Donors were classified as three groups. Bars indicate the mean of each group. Values are mean ± SD. Total *n* = 62 **, *p* < 0.01, ***, *p* < 0.001. NS, no significance (B) Scatter plot of CCL5 (A.U.) versus donor's age. Bars indicate the mean of each group. Values are mean ± SD. Total *n* = 62 **, *p* < 0.01, ***, *p* < 0.001. (C) The relationship of CCL5 and VEGF level normalized with cell number (A. U.). Straight line shows the best fit curve of simple linear regression modelling. *R* = 0.634, *p* < 0.001, Total *n* = 62 (D) CCL5 (A.U.) versus Framingham risk score (FRS). CCL5 level normalized with cell number was plotted with donor's Framingham risk score with age and gender adjustment. Donors were classified as three groups according to the scores. Bars represent the mean of each group. Scores (mean ± SD.) for the low risk group −3–9, 11.1 ± 8.0; medium risk group 10–15, 5.8 ± 5.9; and high risk group 16–21, 2.1 ± 1.4; Total *n* = 62.

As EPC senescence suppressed the secretion of CCL5 in vitro, we wondered if the level of CCL5 in circulating EPCs was affected by age in vivo. To this end, we collected EPCs from 62 donors' peripheral bloods followed by 4 days of culture to determine the circulating EPCs‐derived CCL5 and VEGF levels. As VEGF is a vital growth factor to determine EPC angiogenic activity and viability, we also analysed the levels of VEGF in relation to age. Concordantly, the mean values of CCL5 showed a trend of decrease with the increase of donors' age (Figure 6A), suggesting decreased secretion of CCL5 in seniors' EPCs. We further compared VEGF levels with age classified as three groups ranging from 35 to 85 (Figure 6B). The mean values of VEGF among groups were decreased with the increase of age, indicating the negative relationship between VEGF levels in EPCs and donor's age. The relationship between CCL5 and VEGF was plotted in Figure 6C, showing a close and positive correlation between CCL5 and VEGF (*R* = 0.634 by linear regression modelling, *N* = 62). Since age is an independent risk factor of CVD, we assayed the relationship between CCL5 levels and FRS classified as low, medium and high‐risk groups (ranged from −3 to 9, 10 to 15 and 16 to 21 respectively). The groups with higher FRS were with lower levels of CCL5 (Figure 6D), showing a negative relationship between CCL5 and FRS.

## 4 DISCUSSION

**FIGURE 7:**
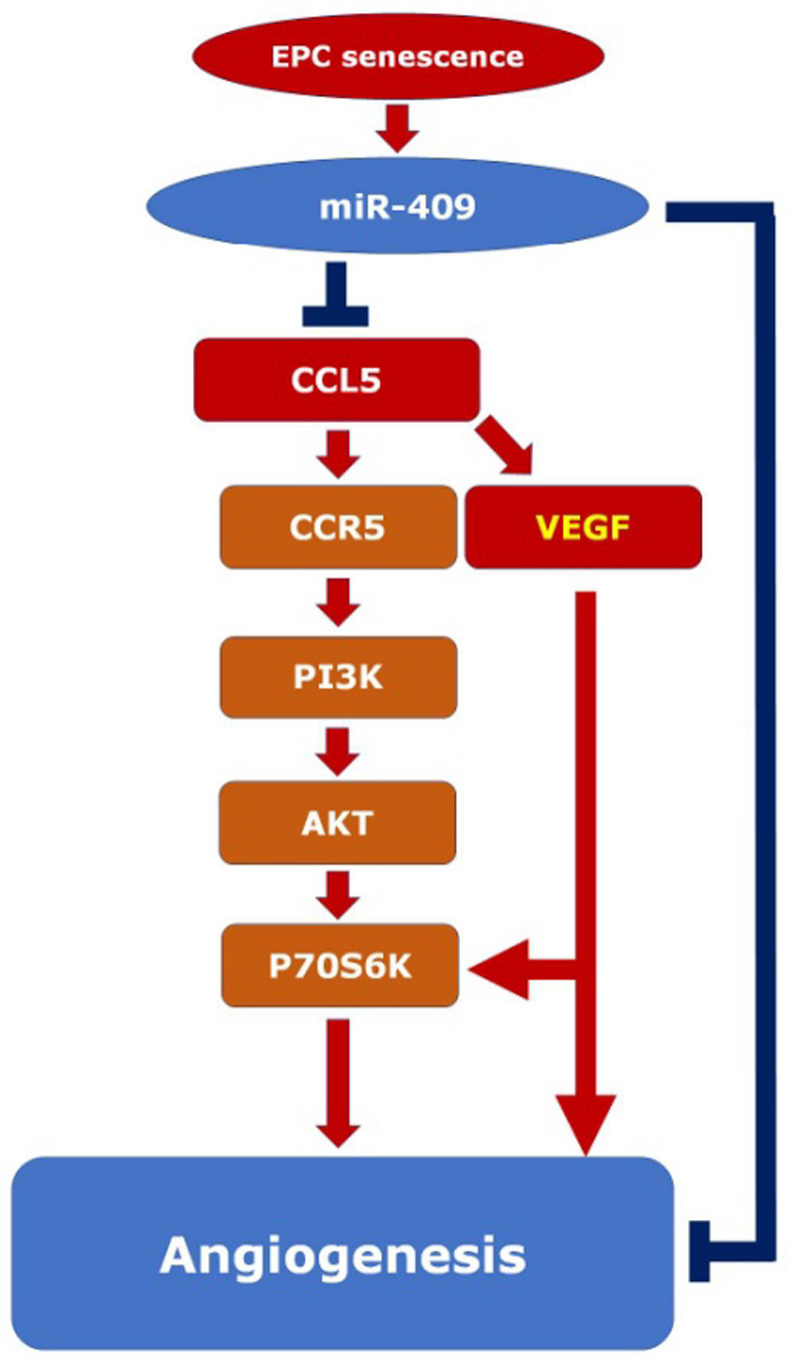
Concept illustration for senescence inducing miR409 expression to down‐regulate CCL5‐mediated EPC angiogenesis. Pathway activation is indicated by arrows, while inhibition is designed by T bars. Senescence up‐regulates the miR‐409 expression, which reduces CCL5 expression and secretion in EPCs. CCL5 increases EPC proliferation, migration, and tube formation (angiogenesis) mainly through CCR5/PI3K/AKT/P70S6K pathway. CCL5 also enhances VEGF secretion which, unlike CCL5, exerts a more balanced enhancement on both EPC proliferation and migration. In our experiment, CCL5 supplement just partially rescued the migration capacity reduced by miR‐409 (Figure 4), indicating the presence of CCL5‐independent pathway (long blue T bar) downstream to miR‐409 (cf. reference 16).

The main finding of the present study (Figure 7) is that senescence decreases the secretion of CCL5 and the level of cognate receptor CCR5 to attenuate angiogenic activity of human EPCs. In mechanistic exploration, we identified that CCL5 played as an autocrine in EPC angiogenesis via activating CCR5/AKT/P70S6K axis. Senescence also induced miR‐409 to decrease the secretion of CCL5 and CCR5 expression, resulting in angiogenic inhibition, while CCL5 was able to induce VEGF expression to counteract miR‐409‐mediated angiogenic inhibition. Our study also implies that miR‐409 impairs angiogenic capacity of EPC through a CCL5‐independent pathway because the addition of CCL5 back to miR‐409 treated EPC only partially rescued the migration capacity (Figure 4). In agreement with this notion, our previous work has demonstrated that miR‐409 also impairs EPC angiogenesis through regulating PP2A/P38 pathway.^16^

The autocrine effect of CCL5 in EPC angiogenesis is similar to a previous study showing that CCL5 plays as a paracrine to promote angiogenesis in human chondrosarcoma cells.^25^ Interestingly, secretion of VEGF induced by CCL5 in chondrosarcoma cell is also via CCR5/AKT/P70S6K axis, albeit through down‐regulation of miR‐200b. Accordingly, CCR5/AKT/P70S6K axis may be a conserved signalling pathway for CCL5 to induce VEGF expression, via different miRNAs in a cell‐type specific manner. In tumour vasculature, the PI3K/AKT/P70S6K pathway is responsible for HIF‐1α to induce VEGF for angiogenesis,^21^, ^23^ suggesting that CCL5 regulates EPC angiogenesis via CCR5/AKT/P70S6K axis.

Our in vitro study demonstrated that replication‐induced EPC senescence led to the expression of miR‐409, which decreased CCL5 level, P70S6K activation and EPC angiogenic capacity. This result was further verified in vivo by the hind limb ischemia experiment showing that CCL5 successfully rescued the angiogenic capacity of old EPCs. It has been reported that miR‐409 acts as a tumour suppressor in breast cancer through down‐regulating AKT by binding to its 3′ UTR.^26^ As P70S6K is the immediately downstream target of AKT, miR‐409 may down‐regulate AKT to decrease P70S6K activity in senescent EPCs. Consistent with the results of the present study, overexpression of miR‐409 also impaired human umbilical endothelial cell (HUVEC) proliferation.^27^

Plasma CCL5 has been evaluated as a risk marker to predict CVD, controversially, both high and low levels of CCL5 have been reported to be associated with disease progression.^28^, ^29^, ^30^ Cavusoglu et al measured the baseline plasma levels of CCL5,^29^ in 389 male patients with coronary angiography. They found that low baseline CCL5 levels were an independent predictor of cardiac mortality, in contrast to a study by Kraaijeveld et al.,^30^ in which plasma levels of CCL5 were elevated in patients with refractory ischemic symptoms versus stabilized patients. Aukrust et al explained that there were several reasons for these apparent discrepancies. First, platelets are the major source of circulating CCL5 levels which are sensitive to the blood sampling protocol. Second, CCL5 level is underestimated because of platelet degranulation in those with active disease, leading to decreased release of CCL5 from platelet ex vivo during storage and thaw procedures. Third, CCL5 bound to heparan sulfate in the vessel wall may be released as the use of heparin is quite common in patients with acute coronary syndrome. In the current study, endogenous CCL5 was harvested from 62 donors' EPCs to exclude the influential platelet source of CCL5. The mean values of both VEGF and CCL5 showed a trend of decrease with the increase of donors' age, consistent with our results of replication‐induced senescence. The relationship of CCL5 and VEGF showed a positive correlation, supporting that CCL5 induces VEGF expression. More importantly, the groups with lower mean values of CCL5 were with higher FRS, indicating that the CCL5 level from EPCs may be a promising biomarker for ageing‐related CVD. Our study, however, was limited by the lack of information regarding exercise levels and diet conditions, which may also have impacts on the EPC number.

In conclusion, our study demonstrated that senescence‐related miR‐409 expression in human EPCs impaired their angiogenesis, at least in part, through the inhibition of CCL5/AKT/P70S6K pathway. Supplement of CCL5 to the senescent EPCs reactivated the pathway and rescued the angiogenic capacity in vitro and in vivo. More importantly, CCL5 secretion from circulating EPCs were reduced along with donors' age and FRS. The data suggest that CCL5 level may be a potentially useful biomarker, and that CCL5 supplement may be a promising treatment strategy for the ageing‐related cardiovascular disorders.

## CLINICAL PERSPECTIVES

1. Reduction of EPC number and function along with age hampers clinical therapies using the cells, however, the detailed underlying mechanisms for the reduction are largely unclear.
2. Senescence significantly up‐regulates miR‐409 expression to decrease the secretion of CCL5, resulting in inhibition on EPC proliferation, migration, and tube formation. Senescence‐induced EPC functional impairment can be rescued by CCL5 and VEGF treatments.
3. In 62 healthy donors, the levels of circulating EPCs‐derived CCL5 are negatively associated with age and Framingham risk score, suggesting CCL5 may serve as a risk predictor for EPC senescence and cardiovascular diseases.

## AUTHOR CONTRIBUTIONS

**Yen‐Hung Chou:** Conceptualization (equal); data curation (equal); investigation (equal); validation (equal). **Yi‐Nan Lee:** Project administration (equal); validation (equal); writing – original draft (equal). **Cheng‐Huang Su:** Project administration (equal); resources (equal); validation (equal). **Hsin‐I Lee:** Data curation (equal); formal analysis (equal); investigation (equal). **Chin‐Ling Hsieh:** Data curation (equal); investigation (equal). **Ting‐Yi Tien:** Project administration (equal); resources (equal). **Chao‐Feng Lin:** Funding acquisition (equal); resources (equal); validation (equal). **Hung‐I Yeh:** Funding acquisition (equal); resources (equal); supervision (equal). **Yih‐Jer Wu:** Funding acquisition (equal); project administration (equal); resources (equal); supervision (equal); writing – review and editing (equal).

## FUNDING INFORMATION

This work was supported by grants from National Science and Technology Council of Taiwan (111‐2314‐B‐715‐014), Ministry of Science and Technology Taiwan (107‐2632‐B‐715‐001), MacKay Memorial Hospital (MMH‐MM‐10703), and MacKay Medical College (RD‐110‐1B‐P013 and 1091B15).

## CONFLICT OF INTEREST STATEMENT

The authors declare that there are no conflicts of interest.

## Data Availability

All supporting data are included within the main article and its supplementary files.
